# Fine‐needle aspiration cytology reduces the frequency of surgeries for malignant salivary gland tumors

**DOI:** 10.1002/cncy.70070

**Published:** 2025-12-19

**Authors:** Marcel Mayer, Sofia Kourou, Marwan Alfarra, Charlotte Laatz, Kevin Hansen, Julia Esser, Hans Nikolaus Caspar Eckel, Kathrin Möllenhoff, Lena Hieggelke, Marianne Engels, Christoph Arolt, Alexander Quaas, Philipp Wolber, Louis Jansen, Lisa Nachtsheim, Jens Peter Klussmann, Sami Shabli

**Affiliations:** ^1^ Department of Otorhinolaryngology Head and Neck Surgery University of Cologne Medical Faculty Cologne Germany; ^2^ Institute of Medical Statistics and Computational Biology University of Cologne Cologne Germany; ^3^ Institute of Pathology University of Cologne Medical Faculty Cologne Germany

**Keywords:** cytology, fine‐needle aspiration cytology, head and neck carcinoma, Milan System for reporting Salivary Gland Cytopathology, salivary gland carcinoma, sonography

## Abstract

**Background:**

Salivary gland tumors are rare and heterogeneous head and neck neoplasms. Preoperative distinction between benign and malignant lesions is challenging because imaging is often insufficient. Fine‐needle aspiration cytology (FNAC) combined with the Milan System for Reporting Salivary Gland Cytopathology (MSRSGC) provides standardized risk stratification and diagnostic guidance; however, its influence on surgical frequency remains insufficiently characterized.

**Methods:**

This retrospective single‐center study included patients with histologically confirmed malignant tumors within the major salivary glands with preoperative FNAC and surgery. The association between MSRSGC category and number of surgeries was evaluated using χ^2^ tests and multivariate Poisson regression.

**Results:**

Overall, 157 patients were included. Those with high‐to‐intermediate‐risk MSRSGC categories (two surgeries: 22.3% vs. one surgery: 77.7%) required significantly fewer surgeries than those with low‐risk/nondiagnostic FNAC (two surgeries: 54.2%/53.3% vs. one surgery: 45.8%/46.7%, *p* < .001). A high‐to‐intermediate risk compared to a nondiagnostic FNAC results was an independent predictor for fewer surgeries in multivariate analysis (incidence rate ratio, 0.875; 95% confidence interval, 0.773–0.990; *p* = .034). True–positive results were most frequent in squamous cell carcinoma, whereas acinic cell and mucoepidermoid carcinomas were often misclassified.

**Conclusions:**

The use of the MSRSGC enables reliable preoperative risk stratification of malignant salivary gland tumors. High‐to‐intermediate‐risk categories (Milan III/IVb/V/VI) were associated with a lower likelihood of multiple surgeries. True–positive FNAC results were most frequent in squamous cell carcinoma and metastatic melanoma, whereas acinic cell, mucoepidermoid, and salivary duct carcinomas were prone to misclassification. Structured FNAC reporting improves diagnostic accuracy and informs personalized surgical planning, reducing interventions and optimizing management.

## Abstract 2

Salivary gland tumors are rare and heterogeneous head and neck neoplasms. Preoperative distinction between benign and malignant lesions is challenging because imaging is often insufficient. Fine‐needle aspiration cytology (FNAC) combined with the Milan System for Reporting Salivary Gland Cytopathology (MSRSGC) provides standardized risk stratification and diagnostic guidance; however, its influence on surgical frequency remains insufficiently characterized. The use of the MSRSGC enables reliable preoperative risk stratification of malignant salivary gland tumors. High‐to‐intermediate‐risk categories (Milan III/IVb/V/VI) were associated with a lower likelihood of multiple surgeries. True–positive FNAC results were most frequent in squamous cell carcinoma and metastatic melanoma, whereas acinic cell, mucoepidermoid, and salivary duct carcinomas were prone to misclassification. Structured FNAC reporting improves diagnostic accuracy and informs personalized surgical planning, reducing interventions and optimizing management.

## INTRODUCTION

Salivary gland tumors represent a rare and heterogeneous subset of head and neck neoplasms, with an incidence of approximately 0.5–1.9 cases per 100,000 individuals annually.^1^, ^2^ Among these are primary salivary gland carcinomas (SGCs), a heterogeneous group comprising 21 distinct histopathological and clinical subtypes with varying biological behavior and prognosis.^3^ In addition to primary malignancies, secondary neoplastic involvement such as metastases from distant solid tumors or lymphomas involving intraparotid lymph nodes may also present within these glands, further complicating the diagnostic workup and process.^4^

Because of the considerable morphological overlap between benign and malignant salivary gland lesions, radiologic imaging alone—despite the widespread use of ultrasound (US), computed tomography (CT), and magnetic resonance imaging (MRI)—is often insufficient to establish a reliable preoperative diagnosis.^5^

Consequently, invasive diagnostic procedures play an important role in the preoperative workup of salivary gland tumors. Therefore, the current European and American clinical guidelines recommend the routine use of fine‐needle aspiration cytology (FNAC) for lesions in the major salivary glands, because of its minimally invasive nature, ease of application, and low complication rate.^6^, ^7^ To enhance the diagnostic clarity and reproducibility of FNAC findings, the Milan System for Reporting Salivary Gland Cytopathology (MSRSGC) was introduced in 2018 and updated in 2023.^8^, ^9^ This system provides a standardized six‐tiered framework for classifying cytological findings according to the implied risk of malignancy, thereby improving communication between cytopathologists and clinicians and facilitating more consistent clinical decision‐making.^10^

Despite the increasing use of FNAC and the MSRSGC, evidence for a clear patient‐centered clinical benefit remains limited. In a large single‐center cohort, it has been demonstrated that preoperative FNAC altered the surgical plan in approximately 19% of parotidectomy cases, primarily by adjusting the extent of resection or adding neck dissection, but did not assess whether this ultimately reduced the overall number of surgeries.^11^ Similarly, subsequent validation studies of the MSRSGC confirmed the diagnostic accuracy and reproducible risk stratification of malignancy within the system, yet did not investigate its clinical impact on surgical rates.^12^ Therefore, in clinical practice, it remains insufficiently studied whether an accurate preoperative diagnosis, achieved through FNAC and precisely classified by the MSRSGC, has a measurable influence on the frequency of surgical interventions.

The key hypothesis of this study was that an accurate preoperative diagnosis using FNAC and applying MSRSGC might lead to a reduction of surgical procedures in cases of malignant salivary gland tumors. To address this question, this study was the first to investigate the relationship between the diagnostic accuracy of FNAC and the frequency of surgery in a large cohort of patients with histologically confirmed malignant salivary gland tumors.

## MATERIALS AND METHODS

This retrospective, single‐center study conducted at the Department of Otorhinolaryngology, Head and Neck Surgery, University Hospital Cologne, Germany, included all patients with preoperative FNAC, subsequent surgical resection, and histopathologically confirmed solid malignant tumor within the major salivary glands between September 1, 2011, and August 31, 2022. Clinical and demographic data, along with cytological and final histopathological results, were collected from the patient charts.

All FNAC procedures were performed under ultrasound guidance using a high‐resolution sonographic system equipped with a linear array transducer operating at 5–12 MHz. Examinations and aspirations were performed by otolaryngologists experienced in head and neck ultrasound. For each salivary gland nodule, one ultrasound‐guided FNAC pass was routinely performed in accordance with the institutional standard operating procedure (SOP). However, it cannot be excluded that in individual cases additional passes were made at the discretion of the puncturing clinician if the target lesion was not sufficiently sampled during the initial attempt. Patients were positioned in supine position, with the performing physician seated to the patient's right side. A 24‐gauge needle, in accordance with the institutional protocol, was used in all procedures and mounted on an empty plastic 10‐cc syringe attached to Cameco syringe holder. Direct smears were prepared by gently spreading the aspirated material between two glass slides. Rapid on‐site evaluation (ROSE) was not performed during the fine‐needle aspiration procedures; instead, all samples were processed and evaluated in the cytopathology laboratory following standard fixation and staining protocols, without immediate adequacy assessment at the time of aspiration. The slides were air‐dried and forwarded to the Institute of Pathology for evaluation. Standard staining protocols included hematoxylin and eosin, May–Grünwald–Giemsa, and, when appropriate, Papanicolaou staining. Cytopathological diagnosis was made by board‐certified (cyto‐)pathologists with special expertise in salivary gland cytology. All cases were reviewed and classified according to the MSRSGC categories.^10^ The Milan categories were grouped according to the risk of malignancy as high‐to‐intermediate‐risk group (Milan III/IVb/V/VI), low‐risk group (Milan II/IVa), and nondiagnostic Group (Milan I) (Table 1). Final histopathological results were classified as low versus high aggressive primary SGC according to the ASCO guidelines or as metastatic disease.^7^

**TABLE 1:** Risk of malignancy according to Milan System for reporting Salivary Gland Cytopathology Category.^13^

Category	ROM (%)
Nondiagnostic, I	15
Nonneoplastic, II	11
AUS, III	30
Neoplasm‐benign, IVa	<3
Neoplasm‐SUMP, IVb	35
SFM, V	83
Malignant, VI	>98

Abbreviations: AUS, atypia of undetermined significance; ROM, risk of malignancy; SFM, suspicious for malignancy; SUMP, salivary gland neoplasm of uncertain malignant potential.

The workflow was performed in accordance with German standards and clinical guidelines. Presentation at the multidisciplinary tumor board was performed for all malignant findings, once the final histopathological diagnosis was available. Frozen section analysis was performed following standard protocols and international guidelines at the time.^7^ Specifically, frozen sections were performed during the initial surgery, if malignancy was suspected or confirmed by preoperative FNAC. This approach ensured that surgical management decisions were guided by histopathological confirmation while adhering to best‐practice oncologic principles.

The decision to perform surgery or resurgery was made in accordance with German standards and the international guidelines for the management of SGC applicable at the time.^7^, ^14^ In cases where a second operation was indicated according to these guidelines, patients typically underwent an ipsilateral neck dissection and a completion revision parotidectomy. Such secondary procedures were performed when the final histopathological assessment after the initial surgery revealed a malignant diagnosis, thereby necessitating therapeutic escalation in line with oncologic treatment principles and based on the recommendations of the multidisciplinary tumor board.

For ancillary studies, no cell blocks were prepared. However, slides from the majority of patients were available and have been digitally archived to enable further evaluation and analyses.

All procedures and analyses were in line with the Declaration of Helsinki. Ethical approval was obtained from the institutional ethics committee of the University of Cologne (Approval code: 24‐1328).

### Statistical methods

Descriptive data were presented as means ± standard deviation for continuous variables and as absolute frequencies and percentages for categorical and dichotomous variables. To evaluate the association between Milan categories and the frequency of surgical interventions, the χ^2^ test was applied. A multivariate Poisson regression analysis was performed to assess the relationship between clinicodemographic parameters and the total number of surgeries. Results were reported as regression coefficients with corresponding 95% confidence intervals (CIs). A *p* value <.05 was considered statistically significant. All statistical analyses were conducted using IBM SPSS Statistics (version 29.0; IBM Corp, Armonk, New York).

## RESULTS

Overall, 1952 salivary gland FNAC cases were identified. Among these, 157 patients with malignant solid tumors within the major salivary glands were included in this study. The mean age of patients was 65.76 (± 16.90) years. Among all patients, 59.9% were male and 40.1% female. The parotid gland was the most frequently affected gland, accounting for 90.4% of cases, followed by the submandibular gland with 9.6%. Regarding laterality, 54.1% of the lesions were located on the right side. Based on the detailed MSRSGC categories, most samples were classified as Milan VI (28.0%), followed by Milan I (19.1%), and Milan V (17.2%). The grouped Milan categories showed that 65.6% of cases fell into the group of high‐to‐intermediate‐risk of malignancy (Milan III/IVb/V/VI), whereas 15.3% were in the low‐risk group (Milan II/IVa), and 19.1% were nondiagnostic (Milan I). Most patients (66.9%) underwent one operation, whereas 33.1% had two operations (Table 2).

**TABLE 2:** Descriptive characteristics.

Variable	Mean/*N* (SD/%)
Age, years	65.8 **(**±16.9)
Gender	
Male	94 (59.9)
Female	63 (40.1)
Gland	
Submandibular gland	15 (9.6)
Parotid gland	142 (90.4)
Side	
Left	72 (45.9)
Right	85 (54.1)
Milan category	
I	30 (19.1)
II	17 (10.8)
III	22 (14.0)
IVa	7 (4.5)
IVb	10 (6.4)
V	27 (17.2)
VI	44 (28.0)
No. of surgeries per patient	
One	105 (66.9)
Two	52 (33.1)

Abbreviations: *N*, number; SD, standard deviation.

The most frequently observed malignant entity was squamous cell carcinoma, accounting for 35.7% of all cases (*n* = 56). This was followed by acinic cell carcinoma and mucoepidermoid carcinoma, each representing 10.2% (*n* = 16) of the cohort. Salivary duct carcinoma comprised 8.3% (*n* = 13), whereas malignant melanoma metastases were present in 7.0% of cases (*n* = 11). Less commonly found entities included salivary adenocarcinoma, not otherwise specified (5.1%), epithelial‐myoepithelial carcinoma (4.5%), basal cell adenocarcinoma (4.5%), and adenoid cystic carcinoma (3.8%). Rare diagnoses such as myoepithelial carcinoma ex pleomorphic adenoma, malignant peripheral nerve sheath tumor, and other metastatic carcinomas were each found in less than 2% of cases (Table 3).

**TABLE 3:** Histopathological diagnosis.

Entity	No. (%)
Total	157
Squamous cell carcinoma	56 (35.7)
Acinic cell carcinoma	16 (10.2)
Mucoepidermoid carcinoma	16 (10.2)
Salivary duct carcinoma	13 (8.3)
Malignant melanoma metastasis	11 (7.0)
Salivary adenocarcinoma, NOS	8 (5.1)
Basal cell adenocarcinoma	7 (4.5)
Epithelial‐myoepithelial carcinoma	7 (4.5)
Adenoid cystic carcinoma	6 (3.8)
Secretory carcinoma	6 (3.8)
Myoepithelial carcinoma ex pleomorphic adenoma	3 (1.8)
Non‐SGC adenocarcinoma metastasis	3 (1.8)
Merkel cell carcinoma metastasis	2 (1.3)
Myoepithelial carcinoma	1 (0.6)
Malignant peripheral nerve sheath tumor	1 (0.6)
High‐grade sarcoma	1 (0.6)

Abbreviations: NOS, not otherwise specified; SGC, salivary gland carcinoma.

The frequency of surgeries was significantly lower among patients with a lesion classified as high‐to‐intermediate‐risk according to the Milan classification compared to those with a lesion classified as low‐risk or nondiagnostic (*p* < .001) (Table 4). In detail, 53.3% of patients with a lesion classified as Milan I and, similarly, 54.2% of patients with a lesion classified as Milan II/IVa underwent two surgeries. When the lesion was correctly classified to have a relevant risk for malignancy (Milan III/IVb/V/VI), two surgeries were performed in only 22.3% (23 of 103) of patients.

**TABLE 4:** Association between Milan categories and number of surgeries.

Milan category	No. of surgeries		Total	% of patients with two surgeries	*p*
	One	Two			
I	14	16	30	53.3	
II/IVa	11	13	24	54.2	<.001
III/IVb/V/VI	80	23	103	22.3	
Total	105	52	157		

To show that the influence of the Milan category as a predictor for the number of surgeries was independent of other variables, a multivariate Poisson regression was performed (Table 5). A true–positive Milan category (III/IVb/V/VI) compared to a nondiagnostic Milan category (I) (incidence rate ratio [IRR], 0.875; 95% confidence interval [CI], 0.773–0.990; *p* = .034), a higher age (IRR, 0.996; 95% CI, 0.993–0.999; *p* = .018), and a metastasis compared to a low‐aggression primary SGC tumor (IRR, 0.777; 95% CI, 0.685–0.881; *p* < .001) were significant independent predictors for a lower number of surgeries. No significant differences were seen between Milan categories II/IVa and Milan category I (IRR, 1.012; 95% CI, 0.867–1.182; *p* = .876). Gender (IRR, 1.000; 95% CI, 0.896–0.990; *p* = 1.116), side (IRR, 1.024; 95% CI, 0.929–1.128; *p* = .634), and tumor location (IRR, 0.908; 95% CI, 0.763–1.080; *p* = .276) were not significantly predicting the number of surgeries.

**TABLE 5:** Multivariate Poisson regression for influence of demographic/clinical data and Milan category on number of surgeries.

Predictor	B (SE)	IRR (95% CI)	*p*
Intercept	0.711 (0.101)	2.034 (1.672–2.481)	<.001
Age, years	–0.004 (0.0015)	0.996 (0.993–0.999)	.018
Gender			
Male	0.0 (0.056)	1.000 (0.896–1.116)	.995
Female	Ref.	Ref.	
Tumor location			
Submandibular gland	–0.097 (0.089)	0.908 (0.763–1.080)	.276
Parotid gland	Ref.	Ref.	
Side			
Left	0.023 (0.049)	1.024 (0.929–1.128)	.634
Right	Ref.	Ref.	
Milan category			
III/IVb/V/VI	–0.134 (0.063)	0.875 (0.773–0.990)	.034
II/IVa	0.012 (0.079)	1.012 (0.867–1.182)	.876
I	Ref.	Ref.	
Aggression status			
Metastasis	–0.252 (0.064)	0.777 (0.685–0.881)	.001
High aggression	–0.031 (0.07)	0.970 (0.845–1.112)	.661
Low aggression	Ref.	Ref.	

Abbreviations: B, logistic regression coefficient; CI, confidence interval; IRR, incidence rate ratio; SE, standard error.

**FIGURE 1:**
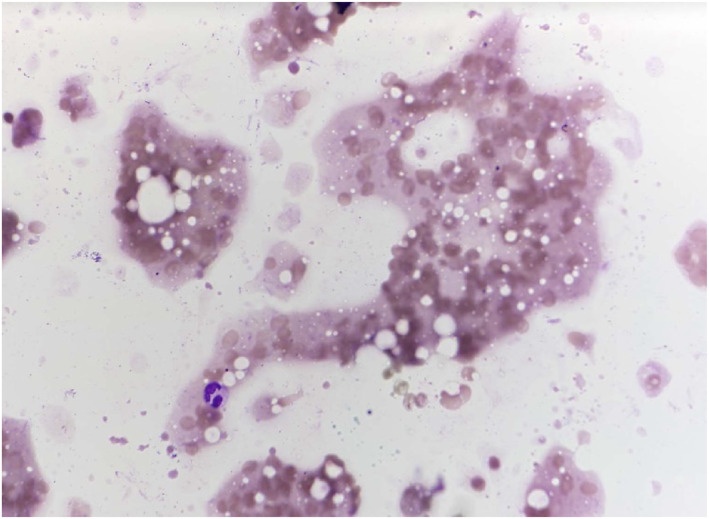
Milan System: Category I, Non‐Diagnostic; May–Grünwald–Giemsa stain 40×. Fine‐needle aspiration smear of a mass lesion of the left parotid gland. The smear contains only peripheral blood; salivary gland epithelium is not included. Histopathological diagnosis was salivary duct carcinoma.

**FIGURE 2:**
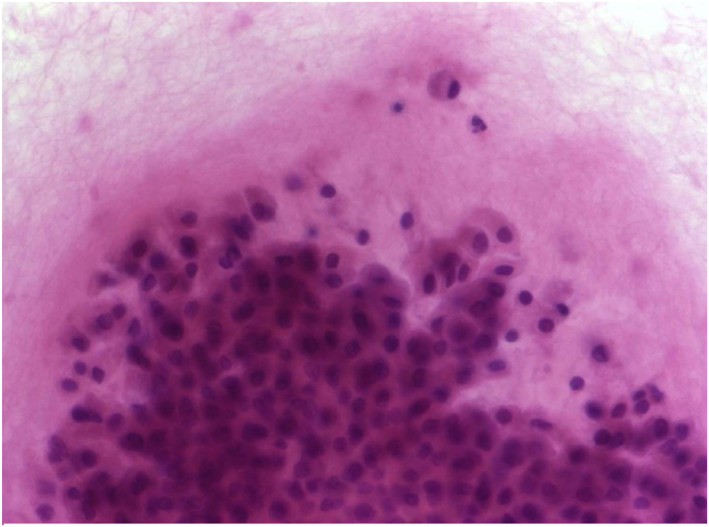
Milan System: Category IVa, Neoplasm, Benign; H & E stain, 60×. Fine‐needle aspiration smear of a mass lesion of the right parotid gland. Large sheet of epithelial cells with oval nuclei, and a dense cytoplasm suggestive of oncocytic differentiation. Cytological diagnosis: benign tumor with oncocytic differentiation, consistent with Warthin tumor. Histopathological diagnosis: false–negative, true diagnosis was mucoepidermoid carcinoma.

**FIGURE 3:**
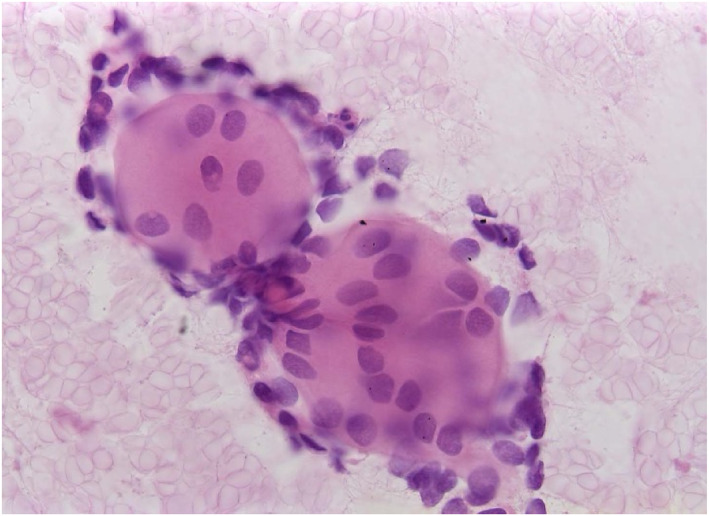
Milan System: Category VI, Malignant; H & E stain 63×. Fine‐needle aspiration smear of a mass lesion of the right parotid gland. Small cells with slightly angular nuclei and an irregular pattern of arrangement. The cells are surrounding spherical bodies of an opaque material staining light red in H & E stain. Diagnosis: malignant solid non–small cell tumor, consistent with adenoid cystic carcinoma. Histopathological diagnosis was adenoid cystic carcinoma.

**FIGURE 4:**
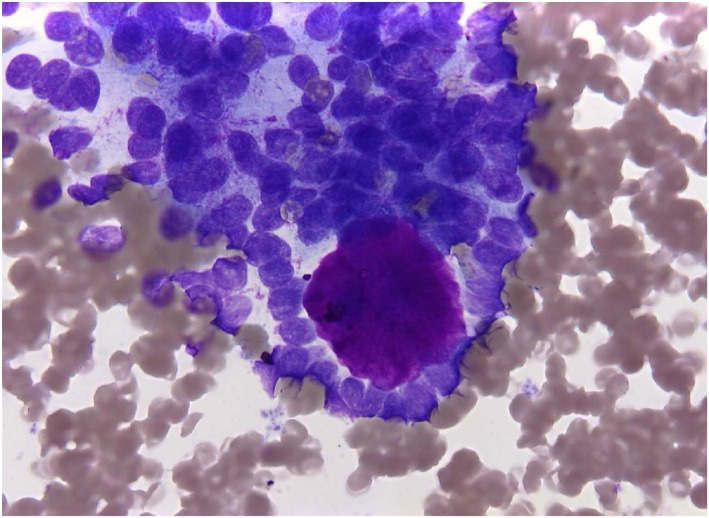
Milan System: Category VI, Malignant; May–Grünwald–Giemsa stain 63×. Fine‐needle aspiration smear of a mass lesion of the right parotid gland. Small cells with slightly angular nuclei and an irregular pattern of arrangement. One large spherical body. Diagnosis: malignant solid non–small cell tumor, consistent with adenoid cystic carcinoma. Histopathological diagnosis was adenoid cystic carcinoma.

Among the five most common malignant salivary gland entities, the distribution of Milan categories based on FNAC showed distinct patterns. For squamous cell carcinoma, 10.7% of cases were classified as nondiagnostic (Milan I), 10.7% of results were false–negative (Milan II/IVa), and the majority (78.6%) true–positive (Milan III/IVb/V/VI). Acinic cell, mucoepidermoid, and salivary duct carcinoma demonstrated with 62.5%, 43.7%, and 53.8%, relatively low frequencies of true‐positive FNAC results (Milan III/IVb/V/VI). On the other hand, metastatic malignant melanoma showed the highest proportion of true–positive results with 90.9% of cases classified as Milan III/IVb/V/VI (Table 6). Figures 1, 2, 3, 4 illustrate representative cytological images corresponding to Milan category I (Figure 1), Milan category IVa (Figure 2), and Milan category VI (Figures 3 and 4), along with the respective cytological descriptions and histopathological diagnoses.

**TABLE 6:** MSRGSC categories according to histopathological diagnosis for the five most frequent entities.

	No. (%)
Squamous cell carcinoma	56 (35.7)
Milan I	6 (10.7)
Milan II/IVa	6 (10.7)
Milan III/IVb/V/VI	44 (78.6)
Acinic cell carcinoma	16 (10.2)
Milan I	3 (18.8)
Milan II/IVa	3 (18.8)
Milan III/IVb/V/VI	10 (62.5)
Mucoepidermoid carcinoma	16 (10.2)
Milan I	6 (37.5)
Milan II/IVa	3 (18.8)
Milan III/IVb/V/VI	7 (43.7)
Salivary duct carcinoma	13 (8.3)
Milan I	3 (23.1)
Milan II/IVa	3 (23.1)
Milan III/IVb/V/VI	7 (53.8)
Malignant melanoma metastasis	11 (7.0)
Milan I	1 (9.1)
Milan II/IVa	0 (0.0)
Milan III/IVb/V/VI	10 (90.9)

Abbreviation: MSRGSC, Milan System for Reporting Salivary Gland Cytopathology.

## DISCUSSION

To the best of our knowledge, this study is the first of its kind providing quantitative evidence that accurate cytological classification of solid malignant tumors in the major salivary glands using FNAC within the MSRSGC framework has a measurable impact on surgical management. Specifically, patients whose FNAC results were categorized as high‐to‐intermediate‐risk of malignancy (Milan III/IVb/V/VI) underwent significantly fewer surgical procedures compared to those with nondiagnostic or low‐risk classifications. Moreover, an intermediate‐to‐high risk Milan category was a significant predictor for a lower number of surgeries even after adjustment for age, tumor type, location, and gender in a multivariate regression model. These findings suggest that FNAC, when applied systematically and interpreted within a standardized cytopathologic framework, can influence treatment pathways and optimize surgical planning, thereby reducing overtreatment and eventually minimizing patient morbidity. Therefore, the ASCO and ESMO guidelines for salivary gland cancer, recommend the routine use of FNAC and the MSRSGC in everyday clinical practice.^6^, ^7^

The demographic characteristics of the study population, including a predominance of male patients and a mean age of 66 years, are consistent with large‐scale epidemiologic studies of salivary gland carcinomas.^2^ Similarly, the distribution of tumor sites, with the parotid gland as the most commonly affected location, aligns with reports highlighting the parotid gland as the principal site for both primary salivary gland malignancies and metastases, especially from cutaneous squamous cell carcinoma.^2^, ^15^, ^16^, ^17^, ^18^, ^19^ The predominance of squamous cell carcinoma as the most frequent histologic diagnosis in our cohort reflects this trend and further underscores the importance of considering metastatic disease in the differential diagnosis of parotid lesions.

The central finding—that patients with correctly categorized high‐to‐intermediate‐risk FNAC results required fewer surgeries—has practical relevance. Previous research has emphasized the high diagnostic accuracy of FNAC when combined with MSRSGC, with reported high sensitivities and specificities ranges up to over 90%.^12^, ^20^, ^21^

However, these studies primarily evaluated diagnostic validity, not clinical consequences. By demonstrating a reduction in surgical interventions, our study provides the first evidence that accurate cytologic classification may translate into tangible benefits in patient care. This reduction is particularly meaningful in parotid surgery, where re‐interventions carry increased risks for facial nerve damage, scar formation, and delayed adjuvant therapy.

It is also worth noting that older patients underwent significantly fewer surgeries, which may be attributed to the increased operative risks associated with advanced age. Consequently, a more conservative, one‐stage approach is often favored in this population to minimize potential complications. Additionally, patients presenting with metastases were less frequently operated on, likely because surgery in these cases often serves primarily for diagnostic confirmation. In the context of metastasectomy, the diagnosis of a malignant primary tumor is usually already established before surgery, allowing for a direct, one‐stage treatment strategy without the need for multiple interventions.

Furthermore, it should be mentioned that the accuracy of FNAC diagnosis varied between different entities, with some being diagnosed correctly less frequently than others are. This variability can partly be attributed to the individual pathological assessment and the potential for misinterpretation by the (cyto‐)pathologist. Moreover, certain entities are inherently difficult to differentiate due to overlapping cytological features, which further complicates accurate diagnosis through FNAC.^21^ Tumors such as mucoepidermoid carcinoma and acinic cell carcinoma were more frequently misclassified into lower‐risk or nondiagnostic categories, which is consistent with prior literature describing these subtypes as cytologically ambiguous. This phenomenon may be partially attributed to the presence of cystic or mucinous degeneration in these tumors, leading to insufficient or misleading cytological samples.^22^, ^23^

In contrast, FNAC demonstrated a high diagnostic accuracy for highly aggressive tumors such as metastatic malignant melanoma and squamous cell carcinoma. This reliability is crucial for guiding therapeutic decisions and optimizing preoperative treatment planning, ensuring timely and appropriate management of these aggressive malignancies.

Future developments in cytopathologic diagnostics, such as immunocytochemistry, molecular profiling, and artificial intelligence‐driven image analysis, hold promise for enhancing diagnostic precision. In thyroid cytology, for example, deep learning models have achieved high accuracy in classifying indeterminate lesions, and similar applications could improve the stratification of challenging salivary gland tumors.^24^, ^25^, ^26^, ^27^, ^28^, ^29^, ^30^ Especially in diagnostically equivocal categories (Milan I–II), these technologies could reduce false–negative rates and improve surgical planning.

Despite the strengths of this study—including a well‐defined cohort, centralized cytopathologic review, and standardized ultrasound‐guided FNAC technique—its retrospective and single‐center nature may limit external generalizability. Multicenter prospective studies with standardized FNAC protocols are needed to validate these findings across varied clinical settings and populations. Additionally, future research should evaluate long‐term oncologic outcomes, including recurrence rates and survival, to determine whether FNAC‐based classification not only reduces surgical interventions but also maintains or even improves disease control. Cost‐effectiveness analyses would also be warranted to quantify the health care savings associated with reduced surgical burden.

In conclusion, this study demonstrates that preoperative FNAC, using the MSRSGC, significantly enhances surgical management of malignant salivary gland tumors by enabling accurate cytological classification and reducing the number of surgeries. Patients with high‐to‐intermediate‐risk FNAC results (Milan III/IVb/V/VI) were significantly less likely to undergo multiple surgeries, independent of age, metastasis status, tumor location, or gender. The diagnostic performance varied among tumor subtypes: squamous cell carcinoma and metastatic melanoma were most accurately classified, whereas acinic cell, mucoepidermoid, and salivary duct carcinomas were frequently misclassified, underscoring limitations of conventional cytology for certain entities. These findings highlight that structured FNAC reporting not only facilitates individualized surgical planning but also has the potential to reduce overtreatment and associated morbidity. Future integration of molecular markers and artificial intelligence‐assisted cytology could further enhance diagnostic accuracy, while prospective studies and cost‐effectiveness analyses are needed to optimize FNAC‐based workflows. Overall, FNAC combined with the MSRSGC represents a vital tool for evidence‐based, personalized treatment strategies in malignant salivary gland disease.

## AUTHOR CONTRIBUTIONS

**Marcel Mayer**: Methodology; visualization; data curation; validation; writing—original draft; writing—review and editing. **Sofia Kourou**: Methodology; validation; visualization; data curation; writing—original draft; writing—review and editing. **Marwan Alfarra**: Writing—review and editing. **Charlotte Laatz**: Writing—review and editing. **Kevin Hansen**: Writing—review and editing. **Julia Esser**: Writing—review and editing. **Hans Nikolaus Caspar Eckel**: Writing—review and editing. **Kathrin Möllenhoff**: Data curation; writing—review and editing. **Lena Hieggelke**: Writing—review and editing. **Marianne Engels**: Writing—review and editing. **Christoph Arolt**: Writing—review and editing. **Alexander Quaas**: Writing—review and editing. **Philipp Wolber**: Writing—review and editing. **Louis Jansen**: Writing—review and editing. **Lisa Nachtsheim**: Writing—review and editing. **Jens Peter Klussmann**: Writing—review and editing. **Sami Shabli**: Writing—review and editing. All authors approved the final submitted manuscript.

## CONFLICT OF INTEREST STATEMENT

The authors declare no conflicts of interest.

## Data Availability

The data sets generated and analyzed during the current study are available from the corresponding author on reasonable request.
